# Supplementary material for: An Environmental Scan and Evaluation of Quality Indicators Across Canadian Kidney Transplant Centers
Source: Can J Kidney Health Dis. 2021 Jun 28;8:20543581211027969. doi: 10.1177/20543581211027969 (PMC8243101; doi:10.1177/20543581211027969)
Supplement: sj-pdf-1-cjk-10.1177_20543581211027969 – Supplemental material for An Environmental Scan and Evaluation of Quality Indicators Across Canadian Kidney Transplant Centers [file sj-pdf-1-cjk-10.1177_20543581211027969.pdf]

Supplementary Table 1: Tool to Evaluate Indicator Strengths and Weaknesses

Source: Stelfox HT and Straus SE. Measuring quality of care: considering measurement frameworks and needs assessment to guide quality indicator development. *J Clin Epidemiol*. 2013; 66: 1320-7.

**QI Dimensions**	**Disagree**			**Neutral**			**Agree**		
Targets important improvements (e.g., large population)	1	2	3	4	5	6	7	8	9
Strong level of evidence for indicator	1	2	3	4	5	6	7	8	9
Performance gap exists	1	2	3	4	5	6	7	8	9
Precisely defined and specified (i.e., reliable)	1	2	3	4	5	6	7	8	9
Feasible to collect data without additional effort	1	2	3	4	5	6	7	8	9
Usable for quality improvement	1	2	3	4	5	6	7	8	9
**Final Rating**	**Unnecessary**			**Supplemental**			**Necessary**		
	1	2	3	4	5	6	7	8	9
