# Supplementary material for: An Environmental Scan and Evaluation of Quality Indicators Across Canadian Kidney Transplant Centers
Source: Can J Kidney Health Dis. 2021 Jun 28;8:20543581211027969. doi: 10.1177/20543581211027969 (PMC8243101; doi:10.1177/20543581211027969)
Supplement: sj-pdf-2-cjk-10.1177_20543581211027969 – Supplemental material for An Environmental Scan and Evaluation of Quality Indicators Across Canadian Kidney Transplant Centers [file sj-pdf-2-cjk-10.1177_20543581211027969.pdf]

An Environmental Scan and Evaluation of Quality Indicators Across Canadian Kidney Transplant Centers

Journal:	*Canadian Journal of Kidney Health and Disease*
Manuscript ID	CJKHD-21-0011.R1
Manuscript Type:	Original Clinical Research Qualitative
Date Submitted by the Author:	12-May-2021
Complete List of Authors:	Glavinovic, Tamara; Sunnybrook Health Sciences Centre, Nephrology<br>Vinson, Amanda; Dalhousie University Faculty of Medicine, Division of Nephrology;<br>Silver, Samuel; Queen's University, Medicine<br>Yohanna, Seychelle; St. Joseph’s Healthcare Hamilton, McMaster University, Nephrology
Keywords:	kidney transplantation,, kidney replacement therapy, transplant outcomes, Quality Indicators, Quality improvement
Abstract:	Background: Kidney transplantation is the optimal treatment for an individual requiring kidney replacement therapy, resulting in improved survival and quality of life while costing the healthcare system less than maintenance dialysis. Achieving and maintaining a kidney transplant requires extensive coordination of several different healthcare services. To improve the quality of kidney transplant care, quality metrics or indicators that encompass all aspects of the individual’s journey to transplant should be measured in a standardized fashion.<br>Objective: To identify, categorize and evaluate strengths and weaknesses of kidney transplant quality indicators currently being used across Canada.<br>Design: An environmental scan of quality indicators being used by kidney organizations and programs.<br>Setting: A 16-member volunteer pan-Canadian panel with expertise in nephrology, transplant and quality improvement.<br>Sample: Transplant programs, as well as provincial transplant and kidney agencies across Canada.<br>Methods: Indicators were first categorized based on the period of transplant care and then using the Institute of Medicine and Donabedian frameworks. A 4-member sub-committee rated each indicator using a modified version of the Delphi consensus technique based on the American College of Physician/Agency for Healthcare Research and Quality criteria. Consensus ratings were subsequently shared with the entire 16-member panel for additional comments.<br>Results: We identified 46 measures related to transplant care across 7 Canadian provinces (9 referral and evaluation, 9 waitlist activity and outcomes, 6 hospitalization for transplant surgery, 12 post-transplant care, 6 organ utilization, 4 living donor). We rated 24 indicators (52%) as necessary to distinguish high quality from poor quality care, most of which measured effective (n=10) or efficient (n=6) care. Only 7/46 (15%) indicators evaluated person-centered or equitable care. Fourteen common indicators were measured by 5 of 7 provinces 10 of which were deemed “necessary”, measuring safe (n=2), effective (n=5), efficient

	(n=2), and equitable (n=1) care.<br>Limitations: The panel lacked patient and allied health representation.<br>Conclusions: There are a large number of kidney transplant quality indicators currently being used in Canada, some of which are common across provinces and focus primarily on measuring effective care. Person-centered and equitable care indicators were lacking, and only half of these indicators were deemed “necessary” for quality improvement. Our results should complement ongoing work to achieve national consensus on the standardization of quality indicators in kidney transplantation.

SCHOLARONE™
Manuscripts

An Environmental Scan and Evaluation of Quality Indicators Across Canadian Kidney Transplant Centers

Tamara Glavinovic MD CM MSc[1], Amanda J Vinson MD SM[2], Samuel A Silver MD MSc[3]*
Seychelle Yohanna MD MSc[4]*

*Both contributed equal supervision to this work

[1]Division of Nephrology, Department of Medicine, Sunnybrook Health Sciences Centre, University of Toronto, Toronto, Ontario, Canada
[2]Division of Nephrology, Department of Medicine, Nova Scotia Health Authority, Dalhousie University, Halifax, Nova Scotia, Canada
[3]Division of Nephrology, Department of Medicine, Kingston Health Sciences Centre, Queen's University, Kingston, Ontario, Canada
[4]Division of Nephrology, Department of Medicine, St. Joseph's Healthcare Hamilton, McMaster University, Hamilton, Ontario, Canada

For Peer Review

Abstract word count: 371

Manuscript word count: 2949~~2693~~

Running title: Kidney transplant quality indicators

Key words: kidney transplantation, kidney replacement therapy, transplant outcomes, quality indicators, quality improvement

Corresponding author:
Tamara Glavinovic
Division of Nephrology, Sunnybrook Health Sciences Centre
1929 Bayview Ave., Rm 378
Toronto, ON, Canada
M4G 3E8
Ph. 416-480-6100 ext. 3863
Fax 416-480-5040
Tamara.glavinovic@sunnybrook.ca

<u>What was known before:</u> Kidney transplant is a cost-effective method of kidney replacement therapy that improves patient survival and quality of life. Caring for individuals pre- and post-transplant requires extensive coordination of care to ensure excellent outcomes, yet it is unclear how quality of care is being measured in Canada.

<u>What this adds:</u> This environmental scan identified 46 kidney transplant quality indicators across Canada. Despite the large number of indicators and some provincial overlap, there were notable gaps in the measurement of person-centered and equitable care. This work provides both a resource of existing quality indicators in kidney transplant and informs future efforts that aim to standardize the measurement of quality in kidney transplant care.

For Peer Review

Abstract

Background: Kidney transplantation is the optimal treatment for an individual requiring kidney replacement therapy, resulting in improved survival and quality of life while costing the healthcare system less than maintenance dialysis. Achieving and maintaining a kidney transplant requires extensive coordination of several different healthcare services. To improve the quality of kidney transplant care, quality metrics or indicators that encompass all aspects of the individual's journey to transplant ~~must~~ should be measured in a standardized fashion.

Objective: To identify, categorize and evaluate strengths and weaknesses of kidney transplant quality indicators currently being used across Canada.

Design: An environmental scan of quality indicators being used by kidney organizations and programs.

Setting: A 16-member volunteer pan-Canadian panel with expertise in nephrology, transplant and quality improvement.

Sample: Transplant programs, as well as provincial transplant and kidney agencies across Canada.

Methods: Indicators were first categorized based on the period of transplant care and then using the Institute of Medicine and Donabedian frameworks. A 4-member sub-committee rated each indicator using a modified version of the Delphi consensus technique based on the American College of Physician/Agency for Healthcare Research and Quality criteria. Consensus ratings were subsequently shared with the entire 16-member panel for additional comments.

Results: We identified 46 measures related to transplant care across 7 Canadian provinces (9 referral and evaluation, 9 waitlist activity and outcomes, 6 hospitalization for transplant surgery, 12 post-transplant care, 6 organ utilization, 4 living donor). We rated 24 indicators (52%) as necessary to distinguish high quality from poor quality care, most of which measured effective (n=10) or efficient (n=6) care. Only 7/46 (15%) indicators evaluated person-centered or equitable care. Fourteen common indicators were measured by 5 of 7 provinces 10 of which were deemed "necessary", measuring safe (n=2), effective (n=5), efficient (n=2), and equitable (n=1) care.

Limitations: The panel lacked patient and allied health representation.

Conclusions: There are a large number of kidney transplant quality indicators currently being used in Canada, some of which are common across provinces and focus primarily on measuring

For Peer Review

effective care. Person-centered and equitable care indicators were lacking, and only half of these indicators were deemed "necessary" for quality improvement. Our results should complement ongoing work to achieve national consensus on the standardization of quality indicators in kidney transplantation.

For Peer Review

Introduction

Kidney transplantation is the optimal treatment for individuals requiring kidney replacement therapy (KRT) as it results in improved survival, enhanced quality of life and reduced healthcare costs compared to maintenance dialysis[1, 2 3, 4]. For an individual with kidney failure, achieving and maintaining a kidney transplant long-term requires extensive oversight,

expertise and coordination of healthcare services across various healthcare sectors (e.g. hospital transplant programs, organ procurement organizations, chronic kidney disease programs, primary care). Many challenges to providing high quality kidney transplant care exist including lack of access demonstrated by variability in referral and transplant rates across programs, prolonged recipient and donor evaluation wait times and stagnant living donor numbers[5-8]. To understand whether transplant programs are providing high quality care and to facilitate improvement activities, a comprehensive view of the individual's journey to transplant and beyond must be translated into measurable quality of care metrics (or quality indicators). However, few examples of how to measure the quality of kidney transplant care exist in Canada [9, 10].

Quality indicators are quantitative or qualitative measures that can be described with different frameworks. The Institute of Medicine (IOM) domains determine if the care provided is safe (free from harm), effective (evidence-based), efficient (limits waste), timely (available when needed), person-centered (focused on the individual), and equitable (equally available)[11]. Quality indicators can also be categorized into the three aspects of quality described in the Donabedian framework: structure measures (the setting in which kidney transplantation occurs, e.g., the presence of a multi-disciplinary team), process

measures (the steps to deliver care, e.g., the proportion of recipients on *pneumocystis jiroveci* (PJP) prophylaxis) and outcome measures (how that care will impact the individual, e.g., graft survival)[12]. Not only can quality indicators determine if predefined benchmarks are met (i.e., quality assurance), but they can also be used by frontline healthcare providers for microsystem quality improvement (i.e., small-scale projects that test iterative changes aiming to improve local performance)[13].

In Canada, transplant programs are somewhat siloed, and little is known about the provincial scope and overlap of existing quality indicators and the different domains of healthcare quality currently being measured in kidney transplantation. The objective of this study was to identify and describe the characteristics of kidney transplant quality indicators currently being utilized in Canada, as well as to highlight their strengths and weaknesses based on the American College of Physicians/Agency for Healthcare Research Quality criteria[14]. We anticipate this work will provide a pan-Canadian resource of existing quality indicators and complement future studies that aim to standardize the use and operationalize definitions for quality indicators used in kidney transplant care.

For Peer Review

Methods

Environmental Scan of Quality Indicators

We collected kidney transplant quality indicators currently in use by provincial agencies and transplant programs within Canada between February 2019 and December 2020. We also asked programs to provide examples of any ongoing or past quality improvement initiatives related to transplant care. We contacted leadership from provincial agencies, nephrology division directors, transplant program administrative directors and transplant nephrologists across Canada. We stopped the environmental scan once we received multiple responses from the majority of provinces and received no further feedback from other provinces.

For Peer Review

We combined quality indicators into a single measure where similarities existed (e.g. proportion of individuals treated with maintenance dialysis referred for transplant was combined with proportion of individuals treated with maintenance dialysis referred for transplant stratified by type of dialysis modality). Next, we organized each indicator into categories of transplant care (e.g. referral and evaluation, waitlist activity and outcomes, hospitalization for transplant surgery, post-transplant care, organ utilization, living donor). We then classified indicators using the IOM (safe, effective, efficient, timely, person-centered, equitable) and Donabedian (structure, process, outcome) frameworks of healthcare quality. We also included balancing measures to evaluate any unintended negative effects that occur with provision of care (i.e., infectious complications of immunosuppression)[15].

Indicator Evaluation

We rated the identified indicators using a modified version of the American College of Physicians/Agency for Healthcare Research and Quality performance measure review criteria, which included the following dimensions (Supplemental Table 1)[13, 16, 17]:

For Peer Review

- Importance: The metric will lead to a measurable and meaningful improvement or there is a clear performance gap.
- Evidence-base: The metric is based on high-quality and high-quantity evidence.
- Measure specifications: The metric can be clearly defined (i.e., numerator and denominator) and reliably captured.
- Feasibility and applicability: The metric is under the influence of healthcare providers and/or the healthcare system, with data collection and improvement activities both feasible and acceptable.

We rated each of these dimensions on a 9-point scale where 1-3 indicated "does not meet criteria," 4-6 "meets some criteria," and 7-9 "meets criteria." Based on these ratings, each indicator then received a final global rating based on its overall ability to distinguish good quality from poor quality[16]. For the global rating, we considered quality indicators as "necessary" if the median rating was 7, 8, or 9 and there was no disagreement by any member. We considered indicators as "unnecessary" if the median rating was 1, 2, or 3 and there was no disagreement by any member. We considered all other indicators as "supplemental."

Overview of the Modified Delphi Process

For Peer Review

We used a modified Delphi approach to evaluate the strengths and weaknesses of the different transplant indicators. This process has been described previously to help classify quality indicators[15, 18-24]. The Delphi panel consisted of a 16-member volunteer national nephrology quality indicator committee with representatives from 7 of 10 provinces. The majority of members possessed advanced training or expertise in quality improvement. From this committee, a 4-person kidney transplant sub-committee was formed.

The quality indicators from the environmental scan were made available to the kidney transplant sub-committee. Each member of the kidney transplant sub-committee separately rated the quality indicators in advance of a teleconference. The sub-committee members then discussed the individual ratings at the teleconference and agreed upon a group rating for each of the American College of Physicians/Agency for Healthcare Research and Quality domains[14]. Next, we circulated the initial group ratings to each sub-committee member for further feedback and to confirm consensus. Last, we shared the final group ratings with the entire 16-member committee, with further discussion of any ratings that differed by ≥3 points. Formal research ethics board review was not required by Queen's University based on the Tri-Council Policy Statement for ethical human research, as the

focus of the study involved quality indicators and not human participants.

Results

Of the 7 provinces that reported currently using kidney transplant quality indicators, our environmental scan identified a total of 46 unique measures (Table 1). Of the 46 indicators, 9 measured transplant referral and evaluation, 9 waitlist activity and outcomes, 6 hospitalization for transplant surgery, 12 post-transplant care, 6 organ utilization and 4 the care of living donors. IOM domains of quality that were covered included safe (n=9, 20%), effective (n=13, 28%), efficient (n=11, 24%), timely (n=6, 13%), person-centered (n=2, 4%), and equitable (n=5, 11%) care. Donabedian categories covered included process (n=19, 41%), outcome (n=14, 30%), and balancing (n=13, 28%). We did not identify any structure measures.

For Peer Review

Table 2 demonstrates the ratings for all 46 kidney transplant indicators. Overall, we rated 24/46 (52%) indicators as "necessary" to distinguish high quality from poor quality care, 22/46 (48%) as "supplemental," and none as "unnecessary. The 24 "necessary" indicators were comprised of 10 process, 9 outcome and 5 balancing measures that focused on safe (n=2/24, 8%), effective (n=10/24, 42%), efficient (n=6/24, 25%), timely (n=4/24, 17%), and equitable (n=2/24, 8%) care. None of the necessary indicators measured person-centered care.

The range of indicators collected by provinces was between 12 to 34 indicators. Thirteen indicators were only being measured by a single province and not measured elsewhere. , Wwe did observed overlap with 14 indicators utilized by at least 5 of 7 provinces. Of these 14 common indicators, there were 4 process measures, 7 outcome measures, 3 balancing measures. These common indicators fell into the IOM categories of safe (n=2), effective (n=5), efficient (n=2), timely (n=1), person-centered (n=2), and equitable (n=2) care. The panel rated 10 of these indicators as "necessary" to distinguish high quality from poor quality care.

Of the 3 provinces that described local quality improvement initiatives, 2 had initiatives related to increasing access to transplant and living donation (e.g., educational and cultural outreach programs). Other initiatives focused on providing safe

For Peer Review

care including vaccination pre-transplant, PJP prophylaxis and cytomegalovirus (CMV)/BK viremia surveillance post-transplant. Only 1 initiative was person-centered and focused on improving the post-transplant experience.

Three common themes emerged during the rating process. First, indicators could be precisely defined and specified, but the definitions were variable and dependent on local practice. For example, for the indicator "time from transplant referral to wait-listing," some programs received most/all required information at the time of referral while other programs must initiate further work-up before wait-listing. Second, the feasibility to collect data or the documentation burden for any given indicator depends largely on data infrastructure or the capabilities of a transplant program's electronic medical record (EMR). This theme was particularly problematic for indicators that relied on education or quality of life (e.g., being informed about transplant as an option). Finally, we rated most indicators (26/46, 57%) as usable for quality improvement (i.e., under the influence of healthcare providers and/or the healthcare system, with data collection and improvement activities both feasible and acceptable). Exceptions included indicators that change too slowly for rapid cycle improvement activities (e.g., kidney transplant prevalence), may not be under the sole control of healthcare providers (e.g., % of

For Peer Review

highly sensitized individuals who receive a transplant), or may not be modifiable (e.g., wait-times by blood type).

Discussion

This environmental scan found 46 kidney transplant quality indicators currently being used across transplant programs and kidney agencies in Canada. The indicators spanned all major periods of transplant care but there were few living donor specific indicators. Using the IOM framework of healthcare quality, the majority of indicators mapped to safe, effective, efficient, and timely care revealing gaps in measuring person-centered and equitable care. There was variation in the indicators being used across provinces, with only 14 indicators common amongst most provinces. These results also provide kidney transplant programs with a selection of 24 "necessary" indicators to distinguish high quality from low quality care, and highlights the need to ensure all domains of healthcare quality and aspects of kidney transplant care are being monitored. Our study complements ongoing work that aims to achieve consensus on which indicators should be used in kidney transplantation across Canada[25].

For Peer Review

Quality indicators in the field of organ transplantation were evaluated in a systematic review by Brett *et al.* and were collected via a Medline, Embase, and Cochrane Central Register of Controlled Trials search from inception until 2017. ~~evaluated in a systematic review by Brett *et al.*, who found a~~ A total of 114 unique indicators were found, 65 of which were related to kidney transplant[10]. There are similarities of note between this systematic review and our study. First, both studies categorized indicators by the IOM and Donabedian frameworks and demonstrate the breadth and quantity of measurement occurring in this field. Second, the majority of indicators were in the quality domains of safety and effectiveness, while a minority measured equity or person-centeredness[10]. Third, few living donor metrics are being tracked (Brett *et al* found no living donor metrics in their search of published articles and our environmental scan found only 4). Important differences are also worth mention. The Brett *et al.* systematic review reported metrics published in the worldwide transplant literature while our study evaluates the current state of quality indicator use in Canada. Brett *et al* classified 23% of their metrics as structure measures, including transplant center volume, appointment no-show rates, program costs, ~~—~~whereas our study identified no structure metrics. While the reasons for this remain unclear, it may be that programs may not fully understand the definitions of various categories of

For Peer Review

quality metrics, which may limit their reporting. Finally, our study used the American College of Physician/Agency for Healthcare Research and Quality criteria to rate the indicators and evaluate their strengths and weaknesses.

Our study also identified several important issues with kidney transplant quality measurement currently occurring in Canada that warrant further attention. Notably, not all areas of transplantation are receiving equal attention, most notably gaps in living kidney donation, which is particularly important because rates of living donation have been stagnant in Canada for some time[26]. Similarly, even though healthcare systems around the world are being redesigned to achieve the quadruple aim (improve population health, experience of care, reduce per capita costs and increase joy in work)[27, 28], our study found no patient reported outcome or experience measures (e.g. functional impairment or quality of life) or cost/resource utilization indicators being routinely measured. We also identified many "necessary" process (n=10) measures, with 70% used by more than one province. This is important to point out as process measures are more easily measured and can demonstrate change more rapidly than outcome measures, making them an important component of rapid-cycle microsystem quality improvement activities[29].

Recently, two Canadian studies attempted to address some of the described shortcomings. The first was a Canadian consensus

For Peer Review

workshop of key stakeholders in transplantation (physicians, patient representatives, allied health) that reviewed potential quality indicators according to predefined criteria (e.g., relevant, actionable, measurable) and provided a recommendation of essential, optional or exclude[25]. The second study was a Delphi panel to achieve consensus on quality indicators used to measure the efficiency of the living kidney donor evaluation[30]. An important observation from the indicators selected in these two studies is that the equity domain of quality continues to lack representation, as confirmed in our environmental scan. In the first workshop described above, indicators classified as equitable care included donor candidates deemed suitable to donate and number of living donor kidney transplants performed. In the living donor Delphi panel, there were no measures of equity. Equitable care metrics are important to develop because of known existing healthcare disparities in minority populations[31, 32]. For example, women, individuals of lower socioeconomic status, and those of African or Indigenous backgrounds have consistently been shown to have a reduced likelihood of transplant referral, wait-listing, and subsequent kidney transplant, emphasizing the need to include patients and vulnerable populations as key stakeholders in an effort to fill the identified gaps[33, 34 35]. Another important observation was the sheer number of indicators that resulted from these two studies

For Peer Review

(54 and 26, respectively), in addition to the 46 indicators we found. Therefore, prioritization of indicators will need to occur as transplant programs may not have the means to undertake this amount of measurement and corresponding quality improvement activities. Our goal with this study is to complement the ongoing efforts by Knoll *et al*, and Garg *et al.* in prioritizing indicators. Ideally, future indicators could be developed and piloted at a local level prior to them being implemented at a national level. A key consideration would be to promote regular re-evaluation of whether these indicators remain valid and suitable for ongoing use and retire those indicators that no longer represent the interests of the various stakeholders. Ideally this re-evaluation could be done at a national level to maintain alignment between independent programs. Our 16-member pan-Canadian quality collaborative is currently working with senior leadership across the country (Canadian Senior Renal Leaders Community of Practice group) to prioritize quality indicators in the various domains of kidney care. We hope this will help focus future local QI initiatives on a few key areas and encourage collaboration between centers embarking on similar initiatives[36].

Our study assists the transplant community with these prioritization activities by identifying 24 "necessary"

https://mc.manuscriptcentral.com/cjkhd

indicators (10 measured by 5 of 7 provinces) that programs are already devoting resources to as a starting point for standardization across Canada. Strengths of this work include the structured approach to indicator categorization and evaluation through applying the IOM and Donabedian frameworks along with the criteria used by American College of Physicians/Agency for Healthcare Research and Quality. Additionally, we involved nephrologists with advanced training and real-life expertise in kidney transplantation and/or quality improvement to ensure relevance to frontline improvement efforts. Our panel included individuals that represent most regions of Canada which helped ensure that quality indicators would be relevant across different healthcare settings.

Limitations deserve mention. First, we did not receive indicators from all kidney transplant centres across Canada and therefore our list of indicators is not exhaustive. Second, our study focused on identifying and rating current indicators without clarifying the operational definitions or how each indicator is being used for quality improvement. Third, we did not automatically include indicators from the Canadian Organ Replacement Register (a nationwide database reporting long-term trends in organ donation and transplantation) unless explicitly stated by programs that they were used for quality improvement activities. Fourth, our evaluation of indicators was not

For Peer Review

anonymized which can result in the group arriving at conclusions primarily because others are doing so (the bandwagon effect)[18]. Fifth, the panel was composed of mainly physicians and thus did not include the valuable perspectives of other stakeholders including administrators, allied health professionals and importantly, patients. Finally, there is subjectivity when using the IOM classification to categorize indicators. For example, the time from transplant referral to receipt of a transplant could be classified as efficient or timely care. The determination of IOM domain should ideally be based on how that indicator is used to drive quality improvement activities.

In summary, we identified 46 kidney transplant quality indicators currently being measured across Canada. There was a paucity of living donor indicators, and we found that the majority of indicators focused on safe, effective, and efficient care, with significant gaps in measuring person-centred and equitable care. Ten of the necessary indicators were common amongst most provinces. We hope this overview serves as a useful guide and starting point that supports ongoing efforts to develop, standardize and curate kidney transplant indicators across Canada.

Acknowledgements: The authors value the support and guidance from Adeera Levin throughout the design and execution of this

For Peer Review

work. The other 12 volunteer quality committee members include: William Beaubien-Souligny, Daniel Blum, Lisa Dubrofsky, Claire Harris, Jay Hingwala, Ali Ibrahim, Amber Molnar, Priya Mysore, Kris Poinen, Sachin Shah, Karthik Tennankore, Alison Thomas. SAS is supported by a Kidney Research Scientist Core Education and National Training (KRESCENT) Program New Investigator Award (co-funded by the Kidney Foundation of Canada, Canadian Society of Nephrology, and Canadian Institutes of Health Research).

For Peer Review

Declaration of Conflicting Interests: TG has participated in consultancy for Paladin Labs Inc. AV has participated in advisory boards/consultancy work for Paladin Labs Inc. SAS has received speaking fees from Sanofi and Baxter Canada. SY has no conflicts of interest relevant to this study. All authors approved the final version of the submitted manuscript. We certify that this manuscript nor one with substantially similar content has been published or is being considered for publication elsewhere.

References

1. Sharma S and Sarnak MJ. Epidemiology: The global burden of reduced GFR: ESRD, CVD and mortality. *Nat Rev Nephrol* 2017; 13: 447-448. 2017/06/20. DOI: 10.1038/nrneph.2017.84.
2. Kostro JZ, Hellmann A, Kobiela J, et al. Quality of Life After Kidney Transplantation: A Prospective Study. *Transplant Proc* 2016; 48: 50-54. 2016/02/27. DOI: 10.1016/j.transproceed.2015.10.058.
3. Canadian Organ Replacement Register Annual Report: Treatment for End-Stage Organ Failure in Canada, 2003 to 2012. . In: Information. CIfH, (ed.). Ottawa, Ontario2014.
4. Rana A, Gruessner A, Agopian VG, et al. Survival benefit of solid-organ transplant in the United States. *JAMA Surg* 2015; 150: 252-259. 2015/01/30. DOI: 10.1001/jamasurg.2014.2038.
5. Naylor KL, Dixon SN, Garg AX, et al. Variation in Access to Kidney Transplantation Across Renal Programs in Ontario, Canada. *Am J Transplant* 2017; 17: 1585-1593. 2017/01/10. DOI: 10.1111/ajt.14133.
6. Kim SJ, Gill JS, Knoll G, et al. Referral for Kidney Transplantation in Canadian Provinces. *J Am Soc Nephrol* 2019; 30: 1708-1721. 2019/08/08. DOI: 10.1681/ASN.2019020127.

For Peer Review

7. Habbous S, Arnold J, Begen MA, et al. Duration of Living Kidney Transplant Donor Evaluations: Findings From 2 Multicenter Cohort Studies. *Am J Kidney Dis* 2018; 72: 483-498. 2018/03/28. DOI: 10.1053/j.ajkd.2018.01.036.
8. Habbous S, Woo J, Lam NN, et al. The Efficiency of Evaluating Candidates for Living Kidney Donation: A Scoping Review. *Transplant Direct* 2018; 4: e394. 2018/12/01. DOI: 10.1097/TXD.0000000000000833.
9. Brett KE, Ertel E, Grimshaw J, et al. Perspectives on Quality of Care in Kidney Transplantation: A Semistructured Interview Study. *Transplant Direct* 2018; 4: e383. 2018/09/21. DOI: 10.1097/TXD.0000000000000820.
10. Brett KE, Ritchie LJ, Ertel E, et al. Quality Metrics in Solid Organ Transplantation: A Systematic Review. *Transplantation* 2018; 102: e308-e330. 2018/03/21. DOI: 10.1097/TP.0000000000002149.
11. *Crossing the Quality Chasm: A New Health System for the 21st Century*. Washington (DC), 2001.
12. Donabedian A. The quality of care. How can it be assessed? *JAMA* 1988; 260: 1743-1748. 1988/09/23. DOI: 10.1001/jama.260.12.1743.
13. Stelfox HT and Straus SE. Measuring quality of care: considering measurement frameworks and needs assessment to guide quality indicator development. *J Clin Epidemiol* 2013; 66: 1320-1327. 2013/09/11. DOI: 10.1016/j.jclinepi.2013.05.018.
14. Agency for Healthcare Research and Quality. AHRQ quality indicators., http://www.qualityindicators.ahrq.gov/ (2012).
15. Greenberg A, Angus H, Sullivan T, et al. Development of a set of strategy-based system-level cancer care performance indicators in Ontario, Canada. *Int J Qual Health Care* 2005; 17: 107-114. 2005/01/25. DOI: 10.1093/intqhc/mzi007.
16. MacLean CH, Kerr EA and Qaseem A. Time Out - Charting a Path for Improving Performance Measurement. *N Engl J Med* 2018; 378: 1757-1761. 2018/04/19. DOI: 10.1056/NEJMp1802595.
17. Mendu ML, Tummalapalli SL, Lentine KL, et al. Measuring Quality in Kidney Care: An Evaluation of Existing Quality Metrics and Approach to Facilitating Improvements in Care Delivery. *Journal of the American Society of Nephrology : JASN* 2020; 31: 602-614. 2020/02/15. DOI: 10.1681/ASN.2019090869.
18. Chia-Chien Hsu BAS. The Delphi Technique: Making Sense of Consensus. *Practical Assessment, Research, and Evaluation* 2007 12.
19. Guttmann A, Razzaq A, Lindsay P, et al. Development of measures of the quality of emergency department care for children using a structured panel process. *Pediatrics* 2006; 118: 114-123. 2006/07/05. DOI: 10.1542/peds.2005-3029.

For Peer Review

20. Kroger E, Tourigny A, Morin D, et al. Selecting process quality indicators for the integrated care of vulnerable older adults affected by cognitive impairment or dementia. *BMC Health Serv Res* 2007; 7: 195. 2007/12/01. DOI: 10.1186/1472-6963-7-195.
21. Lindsay P, Schull M, Bronskill S, et al. The development of indicators to measure the quality of clinical care in emergency departments following a modified-delphi approach. *Acad Emerg Med* 2002; 9: 1131-1139. 2002/11/05. DOI: 10.1111/j.1553-2712.2002.tb01567.x.
22. Bell CM, Brener SS, Comrie R, et al. Quality measures for medication continuity in long-term care facilities, using a structured panel process. *Drugs Aging* 2012; 29: 319-327. 2012/04/03. DOI: 10.2165/11599150-000000000-00000.
23. Morris AM, Brener S, Dresser L, et al. Use of a structured panel process to define quality metrics for antimicrobial stewardship programs. *Infect Control Hosp Epidemiol* 2012; 33: 500-506. 2012/04/06. DOI: 10.1086/665324.
24. Jeffs L, Law MP, Straus S, et al. Defining quality outcomes for complex-care patients transitioning across the continuum using a structured panel process. *BMJ Qual Saf* 2013; 22: 1014-1024. 2013/07/16. DOI: 10.1136/bmjqs-2012-001473.
25. Knoll GA, Fortin MC, Gill J, et al. Measuring quality in living donation and kidney transplantation: moving beyond survival metrics. *Kidney Int* 2020; 98: 860-869. 2020/08/14. DOI: 10.1016/j.kint.2020.07.014.
26. Horvat LD, Shariff SZ, Garg AX, et al. Global trends in the rates of living kidney donation. *Kidney Int* 2009; 75: 1088-1098. 2009/02/20. DOI: 10.1038/ki.2009.20.
27. Berwick DM, Nolan TW and Whittington J. The triple aim: care, health, and cost. *Health Aff (Millwood)* 2008; 27: 759-769. 2008/05/14. DOI: 10.1377/hlthaff.27.3.759.
28. Whittington JW, Nolan K, Lewis N, et al. Pursuing the Triple Aim: The First 7 Years. *Milbank Q* 2015; 93: 263-300. 2015/06/06. DOI: 10.1111/1468-0009.12122.
29. Mountford J and Shojania KG. Refocusing quality measurement to best support quality improvement: local ownership of quality measurement by clinicians. *BMJ Qual Saf* 2012; 21: 519-523. 2012/03/27. DOI: 10.1136/bmjqs-2012-000859.
30. Habbous S, Barnieh L, Litchfield K, et al. A RAND-Modified Delphi on Key Indicators to Measure the Efficiency of Living Kidney Donor Candidate Evaluations. *Clin J Am Soc Nephrol* 2020; 15: 1464-1473. 2020/09/26. DOI: 10.2215/CJN.03780320.
31. Young BA, Hall Y and Rodriguez RA. Health disparities in chronic kidney disease: are we making any progress? *Nephrol News Issues* 2009; 23: 48, 50-41. 2009/05/15.
32. Crews DC, Liu Y and Boulware LE. Disparities in the burden, outcomes, and care of chronic kidney disease. *Curr Opin Nephrol*

For Peer Review

Hypertens 2014; 23: 298-305. 2014/03/26. DOI: 10.1097/01.mnh.0000444822.25991.f6.

33. Patzer RE, Amaral S, Wasse H, et al. Neighborhood poverty and racial disparities in kidney transplant waitlisting. *J Am Soc Nephrol* 2009; 20: 1333-1340. 2009/04/03. DOI: 10.1681/ASN.2008030335.

34. Melk A, Babitsch B, Borchert-Morlins B, et al. Equally Interchangeable? How Sex and Gender Affect Transplantation. *Transplantation* 2019; 103: 1094-1110. 2019/02/13. DOI: 10.1097/TP.0000000000002655.

35. Yeates KE, Cass A, Sequist TD, et al. Indigenous people in Australia, Canada, New Zealand and the United States are less likely to receive renal transplantation. *Kidney Int* 2009; 76: 659-664. 2009/06/26. DOI: 10.1038/ki.2009.236.

36. Erdmann R, Morrin L, Harvey R, et al. Canadian Senior Renal Leaders Community of Practice: Vulnerable Populations With Chronic Kidney Disease-Evidence to Inform Policy. *Can J Kidney Health Dis* 2020; 7: 2054358120930977. 2020/08/13. DOI: 10.1177/2054358120930977.

For Peer Review

An Environmental Scan and Evaluation of Quality Indicators Across Canadian Kidney Transplant Centers

Tamara Glavinovic MD CM MSc[1], Amanda J Vinson MD SM[2], Samuel A Silver MD MSc[3]*
Seychelle Yohanna MD MSc[4]*

*Both contributed equal supervision to this work

[1]Division of Nephrology, Department of Medicine, Sunnybrook Health Sciences Centre, University of Toronto, Toronto, Ontario, Canada
[2]Division of Nephrology, Department of Medicine, Nova Scotia Health Authority, Dalhousie University, Halifax, Nova Scotia, Canada
[3]Division of Nephrology, Department of Medicine, Kingston Health Sciences Centre, Queen's University, Kingston, Ontario, Canada
[4]Division of Nephrology, Department of Medicine, St. Joseph's Healthcare Hamilton, McMaster University, Hamilton, Ontario, Canada

For Peer Review

Abstract word count: 371

Manuscript word count: 2949

Running title: Kidney transplant quality indicators

Key words: kidney transplantation, kidney replacement therapy, transplant outcomes, quality indicators, quality improvement

Corresponding author:
Tamara Glavinovic
Division of Nephrology, Sunnybrook Health Sciences Centre
1929 Bayview Ave., Rm 378
Toronto, ON, Canada
M4G 3E8
Ph. 416-480-6100 ext. 3863
Fax 416-480-5040
Tamara.glavinovic@sunnybrook.ca

What was known before: Kidney transplant is a cost-effective method of kidney replacement therapy that improves patient survival and quality of life. Caring for individuals pre- and post-transplant requires extensive coordination of care to ensure excellent outcomes, yet it is unclear how quality of care is being measured in Canada.

What this adds: This environmental scan identified 46 kidney transplant quality indicators across Canada. Despite the large number of indicators and some provincial overlap, there were notable gaps in the measurement of person-centered and equitable care. This work provides both a resource of existing quality indicators in kidney transplant and informs future efforts that aim to standardize the measurement of quality in kidney transplant care.

For Peer Review

Abstract

Background: Kidney transplantation is the optimal treatment for an individual requiring kidney replacement therapy, resulting in improved survival and quality of life while costing the healthcare system less than maintenance dialysis. Achieving and maintaining a kidney transplant requires extensive coordination of several different healthcare services. To improve the quality of kidney transplant care, quality metrics or indicators that encompass all aspects of the individual's journey to transplant should be measured in a standardized fashion.

Objective: To identify, categorize and evaluate strengths and weaknesses of kidney transplant quality indicators currently being used across Canada.

Design: An environmental scan of quality indicators being used by kidney organizations and programs.

Setting: A 16-member volunteer pan-Canadian panel with expertise in nephrology, transplant and quality improvement.

Sample: Transplant programs, as well as provincial transplant and kidney agencies across Canada.

For Peer Review

Methods: Indicators were first categorized based on the period of transplant care and then using the Institute of Medicine and Donabedian frameworks. A 4-member sub-committee rated each indicator using a modified version of the Delphi consensus technique based on the American College of Physician/Agency for Healthcare Research and Quality criteria. Consensus ratings were subsequently shared with the entire 16-member panel for additional comments.

Results: We identified 46 measures related to transplant care across 7 Canadian provinces (9 referral and evaluation, 9 waitlist activity and outcomes, 6 hospitalization for transplant surgery, 12 post-transplant care, 6 organ utilization, 4 living donor). We rated 24 indicators (52%) as necessary to distinguish high quality from poor quality care, most of which measured effective (n=10) or efficient (n=6) care. Only 7/46 (15%) indicators evaluated person-centered or equitable care. Fourteen common indicators were measured by 5 of 7 provinces 10 of which were deemed "necessary", measuring safe (n=2), effective (n=5), efficient (n=2), and equitable (n=1) care.

Limitations: The panel lacked patient and allied health representation.

Conclusions: There are a large number of kidney transplant quality indicators currently being used in Canada, some of which are common across provinces and focus primarily on measuring

For Peer Review

effective care. Person-centered and equitable care indicators were lacking, and only half of these indicators were deemed "necessary" for quality improvement. Our results should complement ongoing work to achieve national consensus on the standardization of quality indicators in kidney transplantation.

For Peer Review

Introduction

Kidney transplantation is the optimal treatment for individuals requiring kidney replacement therapy (KRT) as it results in improved survival, enhanced quality of life and reduced healthcare costs compared to maintenance dialysis[1, 2 3, 4]. For an individual with kidney failure, achieving and maintaining a kidney transplant long-term requires extensive oversight,

expertise and coordination of healthcare services across various healthcare sectors (e.g. hospital transplant programs, organ procurement organizations, chronic kidney disease programs, primary care). Many challenges to providing high quality kidney transplant care exist including lack of access demonstrated by variability in referral and transplant rates across programs, prolonged recipient and donor evaluation wait times and stagnant living donor numbers[5-8]. To understand whether transplant programs are providing high quality care and to facilitate improvement activities, a comprehensive view of the individual's journey to transplant and beyond must be translated into measurable quality of care metrics (or quality indicators). However, few examples of how to measure the quality of kidney transplant care exist in Canada [9, 10].

Quality indicators are quantitative or qualitative measures that can be described with different frameworks. The Institute of Medicine (IOM) domains determine if the care provided is safe (free from harm), effective (evidence-based), efficient (limits waste), timely (available when needed), person-centered (focused on the individual), and equitable (equally available)[11]. Quality indicators can also be categorized into the three aspects of quality described in the Donabedian framework: structure measures (the setting in which kidney transplantation occurs, e.g., the presence of a multi-disciplinary team), process

For Peer Review

measures (the steps to deliver care, e.g., the proportion of recipients on *pneumocystis jiroveci* (PJP) prophylaxis) and outcome measures (how that care will impact the individual, e.g., graft survival)[12]. Not only can quality indicators determine if predefined benchmarks are met (i.e., quality assurance), but they can also be used by frontline healthcare providers for microsystem quality improvement (i.e., small-scale projects that test iterative changes aiming to improve local performance)[13].

In Canada, transplant programs are somewhat siloed, and little is known about the provincial scope and overlap of existing quality indicators and the different domains of healthcare quality currently being measured in kidney transplantation. The objective of this study was to identify and describe the characteristics of kidney transplant quality indicators currently being utilized in Canada, as well as to highlight their strengths and weaknesses based on the American College of Physicians/Agency for Healthcare Research Quality criteria[14]. We anticipate this work will provide a pan-Canadian resource of existing quality indicators and complement future studies that aim to standardize the use and operationalize definitions for quality indicators used in kidney transplant care.

For Peer Review

For Peer Review

Methods

Environmental Scan of Quality Indicators

We collected kidney transplant quality indicators currently in use by provincial agencies and transplant programs within Canada between February 2019 and December 2020. We also asked programs to provide examples of any ongoing or past quality improvement initiatives related to transplant care. We contacted leadership from provincial agencies, nephrology division directors, transplant program administrative directors and transplant nephrologists across Canada. We stopped the environmental scan once we received multiple responses from the majority of provinces and received no further feedback from other provinces.

We combined quality indicators into a single measure where similarities existed (e.g. proportion of individuals treated with maintenance dialysis referred for transplant was combined with proportion of individuals treated with maintenance dialysis referred for transplant stratified by type of dialysis modality). Next, we organized each indicator into categories of transplant care (e.g. referral and evaluation, waitlist activity and outcomes, hospitalization for transplant surgery, post-transplant care, organ utilization, living donor). We then classified indicators using the IOM (safe, effective, efficient, timely, person-centered, equitable) and Donabedian (structure, process, outcome) frameworks of healthcare quality. We also included balancing measures to evaluate any unintended negative effects that occur with provision of care (i.e., infectious complications of immunosuppression)[15].

Indicator Evaluation

We rated the identified indicators using a modified version of the American College of Physicians/Agency for Healthcare Research and Quality performance measure review criteria, which included the following dimensions (Supplemental Table 1)[13, 16, 17]:

For Peer Review

- Importance: The metric will lead to a measurable and meaningful improvement or there is a clear performance gap.
- Evidence-base: The metric is based on high-quality and high-quantity evidence.
- Measure specifications: The metric can be clearly defined (i.e., numerator and denominator) and reliably captured.
- Feasibility and applicability: The metric is under the influence of healthcare providers and/or the healthcare system, with data collection and improvement activities both feasible and acceptable.

We rated each of these dimensions on a 9-point scale where 1-3 indicated "does not meet criteria," 4-6 "meets some criteria," and 7-9 "meets criteria." Based on these ratings, each indicator then received a final global rating based on its overall ability to distinguish good quality from poor quality[16]. For the global rating, we considered quality indicators as "necessary" if the median rating was 7, 8, or 9 and there was no disagreement by any member. We considered indicators as "unnecessary" if the median rating was 1, 2, or 3 and there was no disagreement by any member. We considered all other indicators as "supplemental."

Overview of the Modified Delphi Process

For Peer Review

We used a modified Delphi approach to evaluate the strengths and weaknesses of the different transplant indicators. This process has been described previously to help classify quality indicators[15, 18-24]. The Delphi panel consisted of a 16-member volunteer national nephrology quality indicator committee with representatives from 7 of 10 provinces. The majority of members possessed advanced training or expertise in quality improvement. From this committee, a 4-person kidney transplant sub-committee was formed.

The quality indicators from the environmental scan were made available to the kidney transplant sub-committee. Each member of the kidney transplant sub-committee separately rated the quality indicators in advance of a teleconference. The sub-committee members then discussed the individual ratings at the teleconference and agreed upon a group rating for each of the American College of Physicians/Agency for Healthcare Research and Quality domains[14]. Next, we circulated the initial group ratings to each sub-committee member for further feedback and to confirm consensus. Last, we shared the final group ratings with the entire 16-member committee, with further discussion of any ratings that differed by ≥3 points. Formal research ethics board review was not required by Queen's University based on the Tri-Council Policy Statement for ethical human research, as the

For Peer Review

focus of the study involved quality indicators and not human participants.

Results

Of the 7 provinces that reported currently using kidney transplant quality indicators, our environmental scan identified a total of 46 unique measures (Table 1). Of the 46 indicators, 9 measured transplant referral and evaluation, 9 waitlist activity and outcomes, 6 hospitalization for transplant surgery, 12 post-transplant care, 6 organ utilization and 4 the care of living donors. IOM domains of quality that were covered included safe (n=9, 20%), effective (n=13, 28%), efficient (n=11, 24%), timely (n=6, 13%), person-centered (n=2, 4%), and equitable (n=5, 11%) care. Donabedian categories covered included process (n=19, 41%), outcome (n=14, 30%), and balancing (n=13, 28%). We did not identify any structure measures.

For Peer Review

Table 2 demonstrates the ratings for all 46 kidney transplant indicators. Overall, we rated 24/46 (52%) indicators as “necessary” to distinguish high quality from poor quality care, 22/46 (48%) as “supplemental,” and none as “unnecessary. The 24 “necessary” indicators were comprised of 10 process, 9 outcome and 5 balancing measures that focused on safe (n=2/24, 8%), effective (n=10/24, 42%), efficient (n=6/24, 25%), timely (n=4/24, 17%), and equitable (n=2/24, 8%) care. None of the necessary indicators measured person-centered care.

The range of indicators collected by provinces was between 12 to 34 indicators. Thirteen indicators were only being measured by a single province and not measured elsewhere. We observed overlap with 14 indicators utilized by at least 5 of 7 provinces. Of these 14 common indicators, there were 4 process measures, 7 outcome measures, 3 balancing measures. These common indicators fell into the IOM categories of safe (n=2), effective (n=5), efficient (n=2), timely (n=1), person-centered (n=2), and equitable (n=2) care. The panel rated 10 of these indicators as “necessary” to distinguish high quality from poor quality care.

Of the 3 provinces that described local quality improvement initiatives, 2 had initiatives related to increasing access to transplant and living donation (e.g., educational and cultural outreach programs). Other initiatives focused on providing safe care including vaccination pre-transplant, PJP prophylaxis and

For Peer Review

cytomegalovirus (CMV)/BK viremia surveillance post-transplant. Only 1 initiative was person-centered and focused on improving the post-transplant experience.

Three common themes emerged during the rating process. First, indicators could be precisely defined and specified, but the definitions were variable and dependent on local practice. For example, for the indicator "time from transplant referral to wait-listing," some programs received most/all required information at the time of referral while other programs must initiate further work-up before wait-listing. Second, the feasibility to collect data or the documentation burden for any given indicator depends largely on data infrastructure or the capabilities of a transplant program's electronic medical record (EMR). This theme was particularly problematic for indicators that relied on education or quality of life (e.g., being informed about transplant as an option). Finally, we rated most indicators (26/46, 57%) as usable for quality improvement (i.e., under the influence of healthcare providers and/or the healthcare system, with data collection and improvement activities both feasible and acceptable). Exceptions included indicators that change too slowly for rapid cycle improvement activities (e.g., kidney transplant prevalence), may not be under the sole control of healthcare providers (e.g., % of

For Peer Review

highly sensitized individuals who receive a transplant), or may not be modifiable (e.g., wait-times by blood type).

Discussion

This environmental scan found 46 kidney transplant quality indicators currently being used across transplant programs and kidney agencies in Canada. The indicators spanned all major periods of transplant care but there were few living donor specific indicators. Using the IOM framework of healthcare quality, the majority of indicators mapped to safe, effective, efficient, and timely care revealing gaps in measuring person-centered and equitable care. There was variation in the indicators being used across provinces, with only 14 indicators common amongst most provinces. These results also provide kidney transplant programs with a selection of 24 "necessary" indicators to distinguish high quality from low quality care, and highlights the need to ensure all domains of healthcare quality and aspects of kidney transplant care are being monitored. Our study complements ongoing work that aims to achieve consensus on which indicators should be used in kidney transplantation across Canada[25].

Quality indicators in the field of organ transplantation were evaluated in a systematic review by Brett *et al.* and were

For Peer Review

collected via a Medline, Embase, and Cochrane Central Register of Controlled Trials search from inception until 2017. A total of 114 unique indicators were found, 65 of which were related to kidney transplant[10]. There are similarities of note between this systematic review and our study. First, both studies categorized indicators by the IOM and Donabedian frameworks and demonstrate the breadth and quantity of measurement occurring in this field. Second, the majority of indicators were in the quality domains of safety and effectiveness, while a minority measured equity or person-centeredness[10]. Third, few living donor metrics are being tracked (Brett *et al* found no living donor metrics in their search of published articles and our environmental scan found only 4). Important differences are also worth mention. The Brett *et al.* systematic review reported metrics published in the worldwide transplant literature while our study evaluates the current state of quality indicator use in Canada. Brett *et al* classified 23% of their metrics as structure measures, including transplant center volume, appointment no-show rates, program costs, whereas our study identified no structure metrics. While the reasons for this remain unclear, it may be that programs may not fully understand the definitions of various categories of quality metrics, which may limit their reporting. Finally, our study used the American College of Physician/Agency for

For Peer Review

Healthcare Research and Quality criteria to rate the indicators and evaluate their strengths and weaknesses.

Our study also identified several important issues with kidney transplant quality measurement currently occurring in Canada that warrant further attention. Notably, not all areas of transplantation are receiving equal attention, most notably gaps in living kidney donation, which is particularly important because rates of living donation have been stagnant in Canada for some time[26]. Similarly, even though healthcare systems around the world are being redesigned to achieve the quadruple aim (improve population health, experience of care, reduce per capita costs and increase joy in work)[27, 28], our study found no patient reported outcome or experience measures (e.g. functional impairment or quality of life) or cost/resource utilization indicators being routinely measured. We also identified many "necessary" process (n=10) measures, with 70% used by more than one province. This is important to point out as process measures are more easily measured and can demonstrate change more rapidly than outcome measures, making them an important component of rapid-cycle microsystem quality improvement activities[29].

Recently, two Canadian studies attempted to address some of the described shortcomings. The first was a Canadian consensus workshop of key stakeholders in transplantation (physicians, patient representatives, allied health) that reviewed potential quality

indicators according to predefined criteria (e.g., relevant, actionable, measurable) and provided a recommendation of essential, optional or exclude[25]. The second study was a Delphi panel to achieve consensus on quality indicators used to measure the efficiency of the living kidney donor evaluation[30]. An important observation from the indicators selected in these two studies is that the equity domain of quality continues to lack representation, as confirmed in our environmental scan. In the first workshop described above, indicators classified as equitable care included donor candidates deemed suitable to donate and number of living donor kidney transplants performed. In the living donor Delphi panel, there were no measures of equity. Equitable care metrics are important to develop because of known existing healthcare disparities in minority populations[31, 32]. For example, women, individuals of lower socioeconomic status, and those of African or Indigenous backgrounds have consistently been shown to have a reduced likelihood of transplant referral, wait-listing, and subsequent kidney transplant, emphasizing the need to include patients and vulnerable populations as key stakeholders in an effort to fill the identified gaps[33, 34 35]. Another important observation was the sheer number of indicators that resulted from these two studies (54 and 26, respectively), in addition to the 46 indicators we found. Therefore, prioritization of indicators will need to occur as transplant programs may not have the means to undertake this amount of measurement and corresponding quality improvement activities. Our goal with this study is to complement the ongoing efforts by Knoll *et al*, and Garg *et al.* in prioritizing indicators. Ideally, future indicators could be

For Peer Review

developed and piloted at a local level prior to them being implemented at a national level. A key consideration would be to promote regular re-evaluation of whether these indicators remain valid and suitable for ongoing use and retire those indicators that no longer represent the interests of the various stakeholders. Ideally this re-evaluation could be done at a national level to maintain alignment between independent programs. Our 16-member pan-Canadian quality collaborative is currently working with senior leadership across the country (Canadian Senior Renal Leaders Community of Practice group) to prioritize quality indicators in the various domains of kidney care. We hope this will help focus future local QI initiatives on a few key areas and encourage collaboration between centers embarking on similar initiatives[36].

Our study assists the transplant community with these prioritization activities by identifying 24 "necessary" indicators (10 measured by 5 of 7 provinces) that programs are already devoting resources to as a starting point for standardization across Canada. Strengths of this work include the structured approach to indicator categorization and evaluation through applying the IOM and Donabedian frameworks along with the criteria used by American College of Physicians/Agency for Healthcare Research and Quality. Additionally, we involved nephrologists with advanced training and real-life expertise in kidney transplantation and/or quality improvement to ensure relevance to frontline improvement

For Peer Review

efforts. Our panel included individuals that represent most regions of Canada which helped ensure that quality indicators would be relevant across different healthcare settings.

Limitations deserve mention. First, we did not receive indicators from all kidney transplant centres across Canada and therefore our list of indicators is not exhaustive. Second, our study focused on identifying and rating current indicators without clarifying the operational definitions or how each indicator is being used for quality improvement. Third, we did not automatically include indicators from the Canadian Organ Replacement Register (a nationwide database reporting long-term trends in organ donation and transplantation) unless explicitly stated by programs that they were used for quality improvement activities. Fourth, our evaluation of indicators was not anonymized which can result in the group arriving at conclusions primarily because others are doing so (the bandwagon effect)[18]. Fifth, the panel was composed of mainly physicians and thus did not include the valuable perspectives of other stakeholders including administrators, allied health professionals and importantly, patients. Finally, there is subjectivity when using the IOM classification to categorize indicators. For example, the time from transplant referral to receipt of a transplant could be classified as efficient or timely care. The

For Peer Review

determination of IOM domain should ideally be based on how that indicator is used to drive quality improvement activities.

In summary, we identified 46 kidney transplant quality indicators currently being measured across Canada. There was a paucity of living donor indicators, and we found that the majority of indicators focused on safe, effective, and efficient care, with significant gaps in measuring person-centred and equitable care. Ten of the necessary indicators were common amongst most provinces. We hope this overview serves as a useful guide and starting point that supports ongoing efforts to develop, standardize and curate kidney transplant indicators across Canada.

Acknowledgements: The authors value the support and guidance from Adeera Levin throughout the design and execution of this work. The other 12 volunteer quality committee members include: William Beaubien-Souligny, Daniel Blum, Lisa Dubrofsky, Claire Harris, Jay Hingwala, Ali Ibrahim, Amber Molnar, Priya Mysore, Kris Poinen, Sachin Shah, Karthik Tennankore, Alison Thomas. SAS is supported by a Kidney Research Scientist Core Education and National Training (KRESCENT) Program New Investigator Award (co-funded by the Kidney Foundation of Canada, Canadian Society of Nephrology, and Canadian Institutes of Health Research).

For Peer Review

Declaration of Conflicting Interests: TG has participated in consultancy for Paladin Labs Inc. AV has participated in advisory boards/consultancy work for Paladin Labs Inc. SAS has received speaking fees from Sanofi and Baxter Canada. SY has no conflicts of interest relevant to this study. All authors approved the final version of the submitted manuscript. We certify that this manuscript nor one with substantially similar content has been published or is being considered for publication elsewhere.

For Peer Review

References

1. Sharma S and Sarnak MJ. Epidemiology: The global burden of reduced GFR: ESRD, CVD and mortality. *Nat Rev Nephrol* 2017; 13: 447-448. 2017/06/20. DOI: 10.1038/nrneph.2017.84.
2. Kostro JZ, Hellmann A, Kobiela J, et al. Quality of Life After Kidney Transplantation: A Prospective Study. *Transplant Proc* 2016; 48: 50-54. 2016/02/27. DOI: 10.1016/j.transproceed.2015.10.058.
3. Canadian Organ Replacement Register Annual Report: Treatment for End-Stage Organ Failure in Canada, 2003 to 2012. . In: Information. CIfH, (ed.). Ottawa, Ontario2014.
4. Rana A, Gruessner A, Agopian VG, et al. Survival benefit of solid-organ transplant in the United States. *JAMA Surg* 2015; 150: 252-259. 2015/01/30. DOI: 10.1001/jamasurg.2014.2038.
5. Naylor KL, Dixon SN, Garg AX, et al. Variation in Access to Kidney Transplantation Across Renal Programs in Ontario, Canada. *Am J Transplant* 2017; 17: 1585-1593. 2017/01/10. DOI: 10.1111/ajt.14133.
6. Kim SJ, Gill JS, Knoll G, et al. Referral for Kidney Transplantation in Canadian Provinces. *J Am Soc Nephrol* 2019; 30: 1708-1721. 2019/08/08. DOI: 10.1681/ASN.2019020127.
7. Habbous S, Arnold J, Begen MA, et al. Duration of Living Kidney Transplant Donor Evaluations: Findings From 2 Multicenter Cohort Studies. *Am J Kidney Dis* 2018; 72: 483-498. 2018/03/28. DOI: 10.1053/j.ajkd.2018.01.036.
8. Habbous S, Woo J, Lam NN, et al. The Efficiency of Evaluating Candidates for Living Kidney Donation: A Scoping Review. *Transplant Direct* 2018; 4: e394. 2018/12/01. DOI: 10.1097/TXD.0000000000000833.
9. Brett KE, Ertel E, Grimshaw J, et al. Perspectives on Quality of Care in Kidney Transplantation: A Semistructured Interview Study. *Transplant Direct* 2018; 4: e383. 2018/09/21. DOI: 10.1097/TXD.0000000000000820.
10. Brett KE, Ritchie LJ, Ertel E, et al. Quality Metrics in Solid Organ Transplantation: A Systematic Review. *Transplantation* 2018; 102: e308-e330. 2018/03/21. DOI: 10.1097/TP.0000000000002149.
11. *Crossing the Quality Chasm: A New Health System for the 21st Century*. Washington (DC), 2001.
12. Donabedian A. The quality of care. How can it be assessed? *JAMA* 1988; 260: 1743-1748. 1988/09/23. DOI: 10.1001/jama.260.12.1743.

For Peer Review

13. Stelfox HT and Straus SE. Measuring quality of care: considering measurement frameworks and needs assessment to guide quality indicator development. *J Clin Epidemiol* 2013; 66: 1320-1327. 2013/09/11. DOI: 10.1016/j.jclinepi.2013.05.018.
14. Agency for Healthcare Research and Quality. AHRQ quality indicators., http://www.qualityindicators.ahrq.gov/ (2012).
15. Greenberg A, Angus H, Sullivan T, et al. Development of a set of strategy-based system-level cancer care performance indicators in Ontario, Canada. *Int J Qual Health Care* 2005; 17: 107-114. 2005/01/25. DOI: 10.1093/intqhc/mzi007.
16. MacLean CH, Kerr EA and Qaseem A. Time Out - Charting a Path for Improving Performance Measurement. *N Engl J Med* 2018; 378: 1757-1761. 2018/04/19. DOI: 10.1056/NEJMp1802595.
17. Mendu ML, Tummalapalli SL, Lentine KL, et al. Measuring Quality in Kidney Care: An Evaluation of Existing Quality Metrics and Approach to Facilitating Improvements in Care Delivery. *Journal of the American Society of Nephrology : JASN* 2020; 31: 602-614. 2020/02/15. DOI: 10.1681/ASN.2019090869.
18. Chia-Chien Hsu BAS. The Delphi Technique: Making Sense of Consensus. *Practical Assessment, Research, and Evaluation* 2007 12.
19. Guttmann A, Razzaq A, Lindsay P, et al. Development of measures of the quality of emergency department care for children using a structured panel process. *Pediatrics* 2006; 118: 114-123. 2006/07/05. DOI: 10.1542/peds.2005-3029.
20. Kroger E, Tourigny A, Morin D, et al. Selecting process quality indicators for the integrated care of vulnerable older adults affected by cognitive impairment or dementia. *BMC Health Serv Res* 2007; 7: 195. 2007/12/01. DOI: 10.1186/1472-6963-7-195.
21. Lindsay P, Schull M, Bronskill S, et al. The development of indicators to measure the quality of clinical care in emergency departments following a modified-delphi approach. *Acad Emerg Med* 2002; 9: 1131-1139. 2002/11/05. DOI: 10.1111/j.1553-2712.2002.tb01567.x.
22. Bell CM, Brener SS, Comrie R, et al. Quality measures for medication continuity in long-term care facilities, using a structured panel process. *Drugs Aging* 2012; 29: 319-327. 2012/04/03. DOI: 10.2165/11599150-000000000-00000.
23. Morris AM, Brener S, Dresser L, et al. Use of a structured panel process to define quality metrics for antimicrobial stewardship programs. *Infect Control Hosp Epidemiol* 2012; 33: 500-506. 2012/04/06. DOI: 10.1086/665324.
24. Jeffs L, Law MP, Straus S, et al. Defining quality outcomes for complex-care patients transitioning across the continuum using a structured panel process. *BMJ Qual Saf* 2013; 22: 1014-1024. 2013/07/16. DOI: 10.1136/bmjqs-2012-001473.

For Peer Review

25. Knoll GA, Fortin MC, Gill J, et al. Measuring quality in living donation and kidney transplantation: moving beyond survival metrics. *Kidney Int* 2020; 98: 860-869. 2020/08/14. DOI: 10.1016/j.kint.2020.07.014.
26. Horvat LD, Shariff SZ, Garg AX, et al. Global trends in the rates of living kidney donation. *Kidney Int* 2009; 75: 1088-1098. 2009/02/20. DOI: 10.1038/ki.2009.20.
27. Berwick DM, Nolan TW and Whittington J. The triple aim: care, health, and cost. *Health Aff (Millwood)* 2008; 27: 759-769. 2008/05/14. DOI: 10.1377/hlthaff.27.3.759.
28. Whittington JW, Nolan K, Lewis N, et al. Pursuing the Triple Aim: The First 7 Years. *Milbank Q* 2015; 93: 263-300. 2015/06/06. DOI: 10.1111/1468-0009.12122.
29. Mountford J and Shojania KG. Refocusing quality measurement to best support quality improvement: local ownership of quality measurement by clinicians. *BMJ Qual Saf* 2012; 21: 519-523. 2012/03/27. DOI: 10.1136/bmjqs-2012-000859.
30. Habbous S, Barnieh L, Litchfield K, et al. A RAND-Modified Delphi on Key Indicators to Measure the Efficiency of Living Kidney Donor Candidate Evaluations. *Clin J Am Soc Nephrol* 2020; 15: 1464-1473. 2020/09/26. DOI: 10.2215/CJN.03780320.
31. Young BA, Hall Y and Rodriguez RA. Health disparities in chronic kidney disease: are we making any progress? *Nephrol News Issues* 2009; 23: 48, 50-41. 2009/05/15.
32. Crews DC, Liu Y and Boulware LE. Disparities in the burden, outcomes, and care of chronic kidney disease. *Curr Opin Nephrol Hypertens* 2014; 23: 298-305. 2014/03/26. DOI: 10.1097/01.mnh.0000444822.25991.f6.
33. Patzer RE, Amaral S, Wasse H, et al. Neighborhood poverty and racial disparities in kidney transplant waitlisting. *J Am Soc Nephrol* 2009; 20: 1333-1340. 2009/04/03. DOI: 10.1681/ASN.2008030335.
34. Melk A, Babitsch B, Borchert-Morlins B, et al. Equally Interchangeable? How Sex and Gender Affect Transplantation. *Transplantation* 2019; 103: 1094-1110. 2019/02/13. DOI: 10.1097/TP.0000000000002655.
35. Yeates KE, Cass A, Sequist TD, et al. Indigenous people in Australia, Canada, New Zealand and the United States are less likely to receive renal transplantation. *Kidney Int* 2009; 76: 659-664. 2009/06/26. DOI: 10.1038/ki.2009.236.
36. Erdmann R, Morrin L, Harvey R, et al. Canadian Senior Renal Leaders Community of Practice: Vulnerable Populations With Chronic Kidney Disease-Evidence to Inform Policy. *Can J Kidney Health Dis* 2020; 7: 2054358120930977. 2020/08/13. DOI: 10.1177/2054358120930977.

For Peer Review

For Peer Review

Table 1: Environmental scan of current Canadian kidney transplant quality indicators
The number in parentheses indicates the number of provinces currently using the listed indicator.

	Donabedian Framework of Healthcare Quality		
**Institute of Medicine Domains of Quality**	**Process**	**Outcome**	**Balancing**
Safe	--% of tacrolimus trough levels in target range (2)<br>--% of recipients on PJP prophylaxis (2)	--eGFR at 1-year post-transplant (7) [a]<br>--% recipients alive after kidney transplantation at 1,3, 5 years (6) [a]<br>-- % of recipients who die with a functioning graft each year (4)	--% of recipients with a surgical complication (1)<br>--% of recipients with a confirmed donor-derived infection (excluding CMV and EBV) (1)<br>--% of recipients hospitalized within 30 days and 1 year of transplant surgery (2)<br>--% of recipients with post-transplant malignancy (2)
Effective	--Cold ischemic time (2)<br>-- Deceased donor conversion rate (%, actual donors/potential donors) (1)	--# of kidneys donated per year per million (subdivided by living, NDD and DCD) (7) [a]<br>--# of transplants per year per million (subdivided by living, pre-emptive, deceased, highly sensitized, paired exchange) (7) [a]<br>--Kidney transplant prevalence (7) [a]<br>--% of living donor transplants that are pre-emptive (1)<br>-- Number of kidney transplants per quarter per program (living donor, deceased donor) (3)	--% of recipients with primary non-function (4)<br>--% of recipients who develop DGF (5) [a]<br>--% of recipients who develop *de novo* DSA (3)<br>--% of recipients with acute rejection in the first-year post-transplant (categorized by type and pathology) (6) [a]<br>--% of recipients with NODAT in the first-year post-transplant (1)<br>-- % recipients who experienced graft loss each year (4)
Efficient	--% of eligible individuals with CKD referred for kidney transplant evaluation (with and without a living donor) (1)<br>--% of eligible individuals on maintenance dialysis referred for kidney transplant evaluation (with and without a living donor) (1)<br>--% of individuals referred for transplant who are waitlisted (3)<br>--% of individuals on waitlist who are active or on-hold (3)<br>--% of organs that are exported, imported, and declined (subdivided by region and % accepted, % cancelled, % used) (4)	--% of living donor candidates who donate (7)[a]<br>--% of individuals referred for transplant who receive a transplant (1)<br>--Length of stay during transplant surgery (1)	--% of individuals who died or who were removed from the waitlist per year (7) [a]<br>--Deceased donor quality calculated by KDPI (3)<br>-- % of potential deceased donors referred for organ donation each year (1)

For Peer Review

Timely	--Time from transplant referral to wait listing (4)<br>--Time from transplant referral (of recipient) to receipt of a living donor transplant (2)<br>--Wait time for deceased donor kidney transplant (subdivided by blood type) (5) [a]<br>-- Wait time for living donor evaluation: from donor contact to approval for donation (2)<br>-- Wait time for living donor evaluation: from donor contact to donation (2)	--% of transplants occurring within the first year of dialysis (3)	
Person Centered	--% of eligible highly sensitized individuals enrolled in highly sensitized program (7) [a]	--% of highly sensitized individuals who receive a transplant (7) [a]	
Equitable	--% of eligible individuals informed about kidney transplant as an option (subdivided by modality) (1)<br>--Waitlist characteristics (total #, blood type, age, sex, allocation points) (7) [a]<br>--# of pairs entered into kidney paired exchange program per million (5) [a]<br>-- # of living donor candidates per year (1)	--% of waitlisted KP individuals who receive a KP transplant (1)	

[a]indicators that were common amongst ≥5/7 of provinces
Abbreviations: CKD=chronic kidney disease, CMV=cytomegalovirus, DCD=donation after circulatory death, DGF=delayed graft function, DSA=donor specific antibody, EBV=Epstein-Barr virus, ESKD=end-stage kidney disease, eGFR=estimated glomerular filtration rate, KDPI=kidney donor profile index, KP=kidney-pancreas, NDD=neurologic determination of death, NODAT=new-onset diabetes after transplant, PJP=*pneumocystis jiroveci* pneumonia

For Peer Review

Table 2: Quality indicators rated by the American College of Physicians/Agency for Healthcare Research and Quality performance measure criteria using a modified Delphi technique
Each domain was rated on a 9-point scale where 1-3 indicated "does not meet criteria," 4-6 "meets some criteria," and 7-9 "meets criteria." After considering and rating each of these domains, the panelists then rated the overall measure (1-3=unnecessary, 4-6=supplemental, 7-9=necessary)

Table 2a: Referral and Evaluation

Indicator	Targets important improvements	Strong level of evidence	Performance gap exists	Precisely defined and specified	Feasible to collect	Usable for QI	Final Rating	Comments
% of eligible individuals informed about transplant as an option (subdivided by modality)	8	7	8	4	4	7	7	Collection of data that accurately captures being informed may be challenging
% of eligible individuals with CKD referred for kidney transplant evaluation (with and without a living donor)	8	7	8	6	6	8	8	Collection of data may be challenging and how a living donor contact is defined varies (i.e., phone vs web portal)
% of eligible individuals on maintenance dialysis referred for kidney transplant evaluation (with and without a living donor)	7	7	8	8	6	8	8	
% of individuals referred for transplant who are waitlisted	7	5	7	7	7	5	7	Process for wait listing may vary between centers
Time from transplant referral to waitlisting	8	4	7	7	7	8	8	Collection of data may be difficult without a centralized system for tracking referrals. Programs sometimes analyzed this indicator in shorter milestones (e.g. time from referral to nephrologist consultation)
Time from transplant referral (of recipient) to receipt of a living donor transplant	8	5	7	7	6	8	8	
% of individuals referred for transplant who receive a transplant	7	7	8	7	7	7	8	
% of living donor transplants that are pre-emptive	8	8	8	8	8	8	8	
# of pairs entered into kidney paired exchange program per million	7	7	8	8	8	7	7	

For Peer Review

Abbreviations: ESKD= end stage kidney disease, CKD=chronic kidney disease

Table 2b: Waitlist activity and outcomes

Indicator	Targets important improvements	Strong level of evidence	Performance gap exists	Precisely defined and specified	Feasible to collect	Usable for QI	Final Rating	Comments
Wait time for deceased donor kidney transplant (subdivided by blood type)	7	7	7	8	8	5	5	Unclear if this metric is modifiable aside from increasing the donor pool
% of eligible highly sensitized individuals enrolled in highly sensitized program	6	5	7	8	8	4	6	
% of highly sensitized individuals who receive a transplant	6	6	7	7	7	4	5	
% of waitlisted KP individuals who receive a KP transplant	7	7	7	8	8	6	6	
% of transplants occurring within the first year of dialysis	8	6	8	8	8	6	6	Influenced by blood type and waitlist criteria, so may be difficult to modify
% of individuals on waitlist who are inactive or on-hold	7	6	7	7	8	6	6	
Waitlist characteristics (total #, blood type, age, sex, allocation points)	5	5	6	8	8	4	5	
% of individuals who died or were removed from the waitlist per year	8	5	6	8	8	8	8	
Number of kidney transplants per quarter per program (living donor, deceased donor)	8	7	8	8	8	9	8	

Abbreviations: KP=kidney-pancreas, cPRA=cumulative panel reactive antibody

Table 2c: Hospitalization for Transplant Surgery

Indicator	Targets important improvements	Strong level of evidence	Performance gap exists	Precisely defined and specified	Feasible to collect	Usable for QI	Final Rating	Comments
% of recipients with primary non-function	8	6	4	7	7	7	7	Rate is low, but it may provide programs with improvement ideas if causes captured

For Peer Review

Cold ischemic time	8	8	8	8	8	8	8	
% of recipients with a surgical complication	7	6	5	5	3	7	6	Important to stratify by type; potentially challenging to collect
% of recipients with a confirmed donor-derived infection (excluding CMV and EBV)	5	4	5	5	3	5	4	May provide insight into donor acceptance practices, but challenging to collect and track
Length of stay during transplant surgery	6	6	8	8	7	7	6	
% of recipients who develop DGF	7	8	7	8	8	7	7	Will need risk adjustment for type of kidneys accepted

Abbreviations: CMV = cytomegalovirus, EBV = ebstein barr virus, DGF = delayed graft function

Table 2d: Post-Transplant

**Indicator**	**Targets important improvements**	**Strong level of evidence**	**Performance gap exists**	**Precisely defined and specified**	**Feasible to collect**	**Usable for QI**	**Final Rating**	**Comments**
% of recipients hospitalized within 30 days and 1 year of transplant surgery	6	5	7	7	4	5	4	Challenging to collect hospitalizations and reasons for hospitalization without an electronic medical record
% of recipients on PJP prophylaxis	6	5	8	7	4	7	6	Challenging to collect without an electronic medical record.
% of tacrolimus trough levels in target range	7	6	7	4	5	7	6	Useful for interventions to improve adherence; challenges with multiple measurements, different assays, and electronic medical records
% of recipients who develop *de novo* donor specific antibody	7	7	5	5	4	6	5	Would require standardized screening protocols (i.e., routine vs triggered) in order to precisely define; costly to implement
% of recipients with acute rejection in the first-year post-transplant	8	7	7	6	6	7	7	May provide insight on immunosuppression/monitoring practices; pathology may be difficult to collect
% of recipients with NODAT in the first-year post-transplant	7	5	5	5	5	5	5	Unclear if this is modifiable.
% of recipients who develop post-transplant malignancy	7	5	5	3	5	5	5	Unclear if modifiable, particularly given the prolonged time frame when cancer can develop; granular

For Peer Review

								information on cancer type also needed
eGFR at one-year post transplant	8	8	7	8	8	6	7	
% of recipients who experienced graft loss each year	8	8	7	8	8	8	8	
% of recipients alive after kidney transplantation at 1,3, 5 years	8	8	7	8	8	8	8	
Kidney transplant prevalence	8	7	7	7	8	4	8	Prevalence does not change rapidly, so difficult to use for improvement
% of recipients who die with a functioning graft each year	6	5	5	8	8	5	6	May be used for quality assurance. Cause of death would be important to ascertain.

Abbreviations: CMV=cytomegalovirus, DGF=delayed graft function, EBV=Epstein-Barr virus, eGFR=estimated glomerular filtration rate, NODAT=new-onset diabetes after transplant, PJP=*pneumocystis jiroveci* pneumonia

Table 2e: Organ Utilization

**Indicator**	**Targets important improvements**	**Strong level of evidence**	**Performance gap exists**	**Precisely defined and specified**	**Feasible to collect**	**Usable for QI**	**Final Rating**	**Comments**
# of kidneys donated per year per million (subdivided by living, NDD and DCD)	8	8	8	8	8	7	7	
% of organs that are exported, imported and declined (subdivided by region and % accepted, % cancelled, % used)	7	5	7	7	7	6	6	
# of transplants per year per million (subdivided by living, pre-emptive, deceased, highly sensitized, paired exchange)	8	8	8	8	8	7	8	Metric is largely impacted by available donor pool
Deceased donor quality calculated by KDPI	4	7	7	6	4	4	6	
% of potential deceased donors referred for organ donation each year	6	6	7	7	6	6	5	
Deceased donor conversion rate (%, actual donors/potential donors)	6	5	7	7	7	5	6	

Abbreviations: DCD=donation after circulatory death, KDPI=kidney donor profile index, KP=kidney-pancreas, NDD=neurologic determination of death

For Peer Review

Table 2f: Living Donor

Indicator	Targets important improvements	Strong level of evidence	Performance gap exists	Precisely defined and specified	Feasible to collect	Usable for QI	Final Rating	Comments
# of living donor candidates per year	9	8	8	4	3	7	6	
% of living donor candidates who donate	8	8	8	8	8	7	8	Requires a standard definition of "candidate"
Wait time for living donor evaluation: from donor contact to approval for donation	8	7	8	8	8	8	8	
Wait time for living donor evaluation: from donor contact to donation	8	5	7	7	6	8	8	

For Peer Review

Supplementary Table 1: Tool to Evaluate Indicator Strengths and Weaknesses

Source: Stelfox HT and Straus SE. Measuring quality of care: considering measurement frameworks and needs assessment to guide quality indicator development. *J Clin Epidemiol*. 2013; 66: 1320-7.

**QI Dimensions**	**Disagree**			**Neutral**			**Agree**		
Targets important improvements (e.g., large population)	1	2	3	4	5	6	7	8	9
Strong level of evidence for indicator	1	2	3	4	5	6	7	8	9
Performance gap exists	1	2	3	4	5	6	7	8	9
Precisely defined and specified (i.e., reliable)	1	2	3	4	5	6	7	8	9
Feasible to collect data without additional effort	1	2	3	4	5	6	7	8	9
Usable for quality improvement	1	2	3	4	5	6	7	8	9
**Final Rating**	**Unnecessary**			**Supplemental**			**Necessary**		
	1	2	3	4	5	6	7	8	9

For Peer Review

CJKHD-21-0011

An Environmental Scan and Evaluation of Quality Indicators Across Canadian Kidney Transplant Centers

Word count: 371

Contexte : La transplantation rénale constitue le traitement optimal pour une personne nécessitant une thérapie de remplacement rénal. La greffe améliore la survie et la qualité de vie du patient, tout en s'avérant moins coûteuse pour le système de santé que la dialyse d'entretien. La réussite et le maintien d'une transplantation rénale requièrent la parfaite coordination de plusieurs services de santé différents. L'amélioration des soins entourant la greffe passe donc par la mesure normalisée des indicateurs de qualité qui englobent tous les aspects du cheminement du patient vers la transplantation.

Objectifs : Identifier, classer et évaluer les forces et faiblesses des indicateurs actuellement utilisés au Canada pour mesurer la qualité des soins entourant la transplantation rénale.

Type d'étude : Analyse contextuelle des indicateurs de la qualité utilisés par les organismes et programmes de néphrologie.

For Peer Review

Cadre : Un comité bénévole pancanadien composé de 16 personnes détenant une expertise en néphrologie, en transplantation et en amélioration de la qualité.

Échantillon : Les programmes de transplantation et les organismes provinciaux de transplantation et de néphrologie partout au Canada.

Méthodologie : Les indicateurs ont d'abord été catégorisés selon le moment des soins, puis avec les modèles de l'Institute of Medicine et de Donabedian. Un sous-comité de quatre personnes a évalué les indicateurs à l'aide d'une version modifiée de la méthode Delphi basée sur les critères de l'American College of Physicians/Agency for Healthcare Research and Quality. Les évaluations consensuelles ont ensuite été partagées avec les 16 membres du comité afin de recueillir d'autres commentaires.

Résultats : Nous avons recensé 46 mesures liées aux soins de transplantation dans sept provinces canadiennes (9 aiguillages et évaluations, 9 activités et résultats liés à la liste d'attente, 6 hospitalisations en vue d'une greffe, 12 soins post-transplantation, 6 utilisations d'organes et 4 donneurs vivants). Nous avons évalué 24 indicateurs (52 %) comme étant nécessaires pour départager les soins de haute qualité des soins de mauvaise qualité, la plupart mesurant l'efficacité (n = 10) ou l'efficience (n = 6). Seuls

For Peer Review

7 indicateurs sur 46 (15 %) évaluaient des soins équitables ou axés sur la personne. Quatorze indicateurs communs étaient mesurés par cinq des sept provinces. Parmi eux, dix mesurant des soins sûrs (n = 2), efficaces (n = 5), efficients (n = 2) et équitables (n = 1) ont été jugés « nécessaires ».

Limites : Le comité manquait de représentation parmi les patients et les professionnels paramédicaux.

Conclusion : Un grand nombre d'indicateurs de la qualité de la transplantation rénale sont utilisés au Canada, certains sont communs à plusieurs provinces et mettent principalement l'accent sur l'efficacité des soins. Mais seulement la moitié de ceux-ci sont jugés « nécessaires » pour améliorer la qualité. De plus, des indicateurs quant aux soins équitables et axés sur la personne manquaient. Nos résultats devraient compléter les travaux en cours visant l'obtention d'un consensus national sur la normalisation des indicateurs de qualité en transplantation rénale.

For Peer Review
